# Comparison of spectral estimators for characterizing fractionated atrial electrograms

**DOI:** 10.1186/1475-925X-12-72

**Published:** 2013-07-16

**Authors:** Edward J Ciaccio, Angelo B Biviano, Hasan Garan

**Affiliations:** 1 Department of Medicine – Division of Cardiology, Columbia University Medical Center, 177 Fort Washington Avenue, New York, USA

**Keywords:** Atrial fibrillation, Dominant frequency, Paroxysmal, Persistent, Spectral estimation

## Abstract

**Background:**

Complex fractionated atrial electrograms (CFAE) acquired during atrial fibrillation (AF) are commonly assessed using the discrete Fourier transform (DFT), but this can lead to inaccuracy. In this study, spectral estimators derived by averaging the autocorrelation function at lags were compared to the DFT.

**Method:**

Bipolar CFAE of at least 16 s duration were obtained from pulmonary vein ostia and left atrial free wall sites (9 paroxysmal and 10 persistent AF patients). Power spectra were computed using the DFT and three other methods: 1. a novel spectral estimator based on signal averaging (NSE), 2. the NSE with harmonic removal (NSH), and 3. the autocorrelation function average at lags (AFA). Three spectral parameters were calculated: 1. the largest fundamental spectral peak, known as the dominant frequency (DF), 2. the DF amplitude (DA), and 3. the mean spectral profile (MP), which quantifies noise floor level. For each spectral estimator and parameter, the significance of the difference between paroxysmal and persistent AF was determined.

**Results:**

For all estimators, mean DA and mean DF values were higher in persistent AF, while the mean MP value was higher in paroxysmal AF. The differences in means between paroxysmals and persistents were highly significant for 3/3 NSE and NSH measurements and for 2/3 DFT and AFA measurements (p<0.001). For all estimators, the standard deviation in DA and MP values were higher in persistent AF, while the standard deviation in DF value was higher in paroxysmal AF. Differences in standard deviations between paroxysmals and persistents were highly significant in 2/3 NSE and NSH measurements, in 1/3 AFA measurements, and in 0/3 DFT measurements.

**Conclusions:**

Measurements made from all four spectral estimators were in agreement as to whether the means and standard deviations in three spectral parameters were greater in CFAEs acquired from paroxysmal or in persistent AF patients. Since the measurements were consistent, use of two or more of these estimators for power spectral analysis can be assistive to evaluate CFAE more objectively and accurately, which may lead to improved clinical outcome. Since the most significant differences overall were achieved using the NSE and NSH estimators, parameters measured from their spectra will likely be the most useful for detecting and discerning electrophysiologic differences in the AF substrate based upon frequency analysis of CFAE.

## Background

The dominant frequency (DF) is an important measure for assessing complex fractionated atrial electrograms (CFAE) in patients with atrial fibrillation (AF) [1-3]. CFAE are defined as electrograms composed of ≥ 2 deflections per cycle without return to the isoelectric interval, or having a cycle length < 120 ms, as averaged over a 10 s recording period [4]. Studies have shown that use of radiofrequency catheter ablation at CFAE regions may be assistive in eliminating the arrhythmogenic substrate by which AF is inducible and perpetuates [4]. CFAE can be analyzed computationally using time domain [5,6] or frequency domain methods [2,3,7]. However, time and frequency methods of digital signal analysis do not have equal robustness. When amplitude varies randomly, as can be common in CFAE recordings, time domain methods lose performance for characterizing AF electrograms, while frequency-domain methods remain stable [8].

The DF can be defined as the tallest fundamental spectral peak within the electrophysiologic range [7,9]. The inverse of this frequency is, to a first approximation, the atrial activation rate [10,11]. For DF calculation, electrograms are traditionally preprocessed by digital bandpass filtering and rectification of the signal, followed by additional low pass filtering [3,12,13]. The preprocessing step generates a smoothed signal that is proportional to the amplitude of the high-frequency components in the original atrial electrogram [12,13]. This enhances the presence of periodicity or nonperiodicity in the waveform [14] by transforming sharp biphasic deflections into sinusoidal-like shapes, with the result that the fundamental frequency is more likely to correspond to the atrial rate [10,15]. However, amplitude and frequency variability can significantly degrade DF measurement of the preprocessed signals when the discrete Fourier transform (DFT) is used [15-17], and thus the preprocessing step is sometimes skipped [18].

Recently, a novel spectral estimator (NSE), derived from a mathematical transform having an orthogonal, data-driven basis [19], was applied to CFAE for frequency analysis [19,20]. Since the basis is data-driven, it is estimative of all digital signal components, including the sharp biphasic deflections commonly present in electrograms, without the need to resort to the distortive preprocessing step that is often used for DFT analysis. Although subharmonics and cross-terms are not significant for DFT analysis because the Fourier basis is sinusoidal and antisymmetric, they may be significant in NSE spectra [21]. In this study, spectral estimators derived from the aforementioned signal averaging transform were implemented to compare performance versus the DFT for discerning differences in paroxysmal versus persistent AF data.

## Method

### Clinical data and the electrophysiology procedure

Atrial electrograms were recorded in 19 patients referred to the Columbia University Medical Center cardiac electrophysiology laboratory for catheter ablation of AF. These recordings were obtained for prospective analysis as approved by the Institutional Review Board, but analyzed retrospectively for this study. Nine patients had documented clinical paroxysmal AF, and normal sinus rhythm was their baseline cardiac rhythm in the electrophysiology laboratory. Atrial fibrillation was induced in these patients by burst atrial pacing from the coronary sinus or right atrial lateral wall, and was required to persist for ≥10 minutes prior to data collection. Ten other patients had longstanding persistent AF without interruption for from several months to years prior to their catheter mapping and ablation procedure. Bipolar atrial mapping was performed with a NaviStar ThermoCool catheter, 7.5F, 3.5 mm tip, with 2 mm spacing between bipoles (Biosense-Webster Inc, Diamond Bar, CA, USA). The electrogram signals were acquired using the General Electric CardioLab system (GE Healthcare, Waukesha, WI), and filtered at acquisition from 30-500 Hz with a single-pole bandpass filter to remove baseline drift and high frequency noise. The filtered signals were sampled at 977 Hz and stored. Although the bandpass high end was slightly above the Nyquist frequency, negligible CFAE signal energy resides in this frequency range [9].

Only signals identified as CFAEs by two cardiac electrophysiologists were included for analysis. CFAE recordings of at least 16 s in duration were obtained from two sites outside the ostia of each of the four pulmonary veins and from two left atrial free wall sites, one in the mid posterior wall, and another on the anterior ridge at the base of the left atrial appendage. The mapping catheter was navigated at these locations until a CFAE site was identified. A total of 204 digitized sequences – 90 from paroxysmal and 114 from longstanding AF patients, all meeting the criteria for CFAE, were selected for this study and included for computational analysis. As in previous studies, to standardize the morphological characteristics, all digital CFAE signals were normalized to mean zero and unity variance (average level = 0 volts, standard deviation and variance = 1) [19-21]. Short signal segments were considered to also be approximately mean zero and unity variance, so that the autocovariance and autocorrelation functions were considered to be equivalent.

### Power spectral generation using ensemble averaging

**Figure 1:**
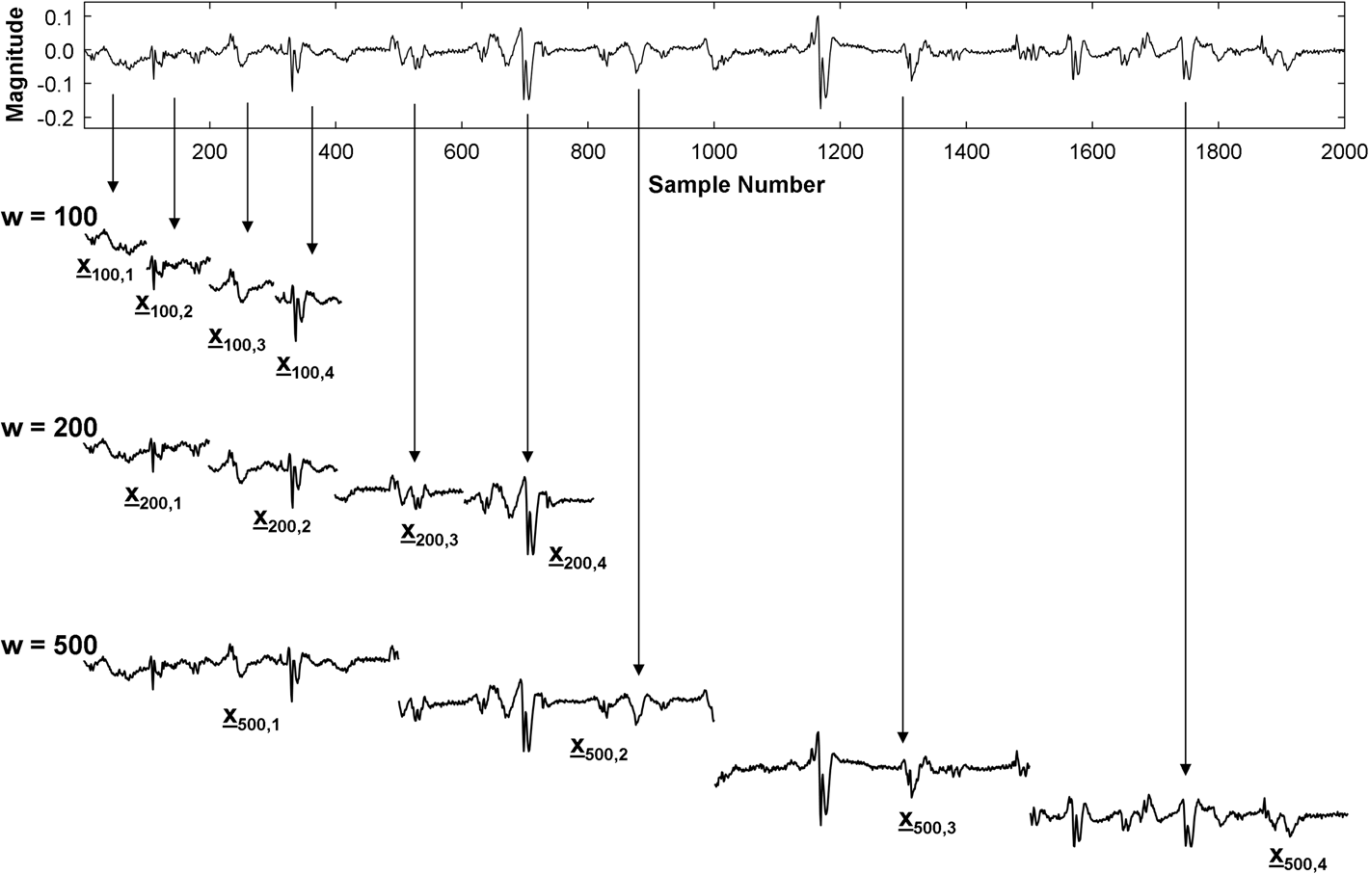
**A. CFAE and its first four signal segments with w = 100, 200, and 500.** Arrows show some of the relationships of the signal segments with respect to the original signal at top.

NSE spectra were generated as described in detail previously [7,9,19-21] and mentioned briefly here. In the equations below, underline signifies a vector, a capital letter indicates a matrix, and the first subscript denotes the vector or matrix dimension. A signal ${\underline{x}}_{N}$ of length N can be divided into n segments of length w for ensemble averaging:

(1) $${\underline{e}}_{w}=\frac{1}{n}\sum_{i}{\underline{x}}_{w,i},\;i=1\;\mathrm{to}\;n$$

where ${\underline{e}}_{w}$ is the ensemble average vector of length w,

(2) $${\underline{x}}_{N}=\left[\begin{array}{c} {\underline{x}}_{w,1}\\ {\underline{x}}_{w,2}\\ ...\\ {\underline{x}}_{w,n} \end{array}\right]$$

and:

(3) $$n=\mathrm{int}\frac{N}{w}$$

where ‘int’ is the integer function (the real number is rounded down). Examples of the summation of signal segments of lengths w = 100, 200, and 500 are shown in Figure 1. If the signal is 2000 discrete sample points long as in Figure 1, then to form the ensemble average, according to Eq. 3 there would be n = 20 segments of length w = 100, n = 10 segments of length w = 200, and n = 4 segments of length w = 500. The first four segments that are extracted from the signal are shown, which are all of the segments when w = 500. By summing all n segments, and dividing by n, the ensemble average of signal segments of length w is obtained. The ensemble average power is then given by:

(4a) $$P_{w}=\frac{1}{w}\cdot {\underline{e}}_{w}^{T}\cdot {\underline{e}}_{w}$$

(4b) $$=\frac{1}{n^{2}w}\;\sum_{i}{\underline{x}}_{w,i}^{T}\;\cdot \;\sum_{j}\;{\underline{x}}_{w,j}\;i=1\;\mathrm{to}\;n,\;j=1\;\mathrm{to}\;n$$

(4c) $$=\frac{1}{N\;w}\;\sum_{i}\sum_{j}\;{\underline{x}}_{w,i}^{T}\;\cdot \;{\underline{x}}_{w,j}\;i=1\;\mathrm{to}\;n,\;j=1\;\mathrm{to}\;n$$

**Figure 2:**
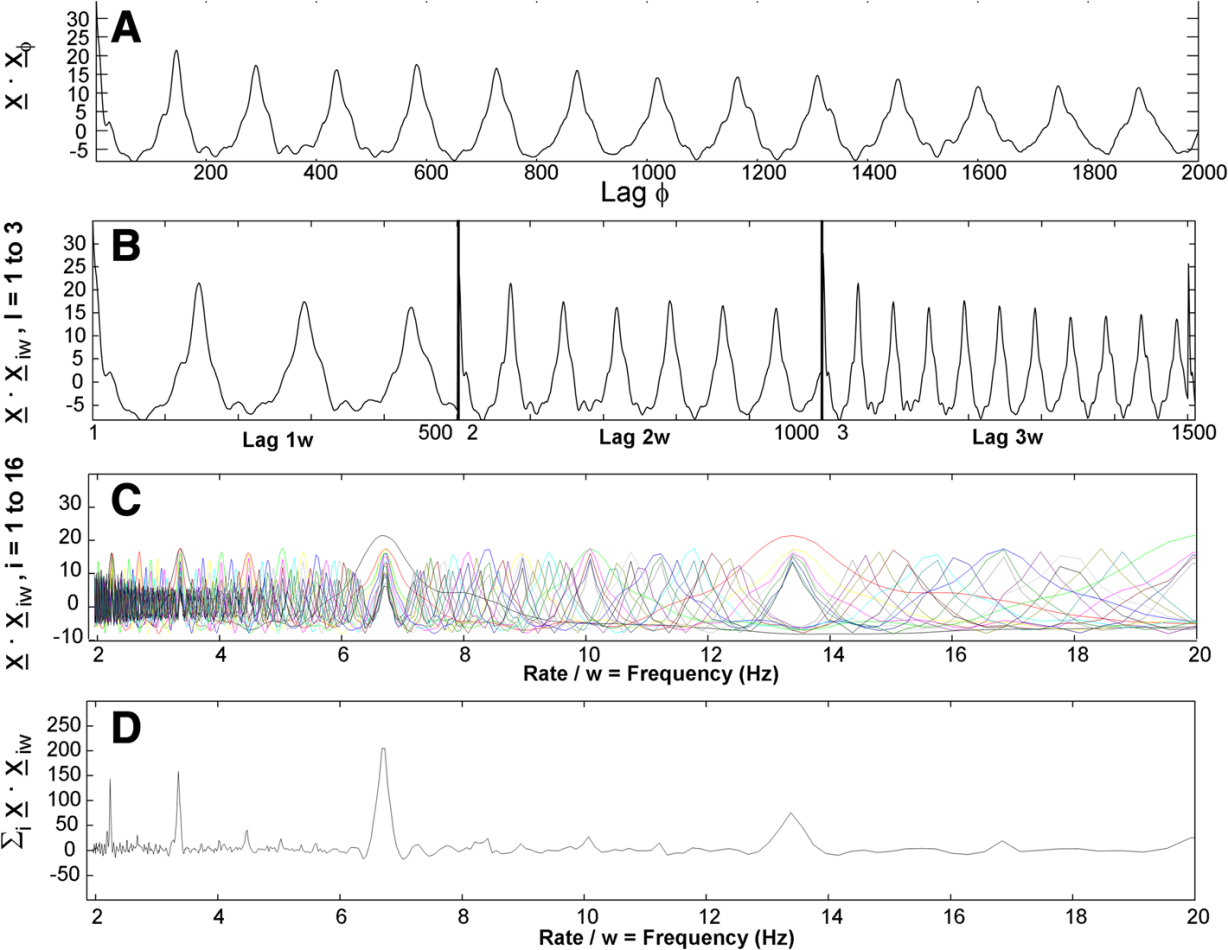
**Mechanism by which the average autocorrelation spectrum sharpens as n lags are added to form the average. A**. The autocorrelation function. **B**. Lag vectors. The frequency range shown in panels **C** and **D**, 2-20 Hz, corresponds to w = 500–50 sample points for the signal digitized at 977 Hz.

It is evident from Eq. 4c that the ensemble average power is related to the autocorrelation function. Suppose a segment length w = 500 and signal length N = 2000 as described in Figure 1. Then from Eq. 3, n = 4. Since in Eq. 4c the indices will then be given by i = 1 to 4 and j = 1 to 4, the phase lags between the four signal segments are given by:

(5) $$\left[\begin{array}{l} \begin{array}{cccc} i\backslash j & 1 & 2 & 3 \end{array}\;4\\ \begin{array}{cccc} 1 & \;0 & 1 & 2 \end{array}\;3\\ \begin{array}{cccc} 2 & \;1 & 0 & 1 \end{array}\;2\\ \begin{array}{cccc} 3 & \;2 & 1 & 0 \end{array}\;1\\ \begin{array}{cccc} 4 & \;3 & 2 & 1 \end{array}\;0 \end{array},\;\right]\;=\;\mathrm{lags}\;\mathrm{matrix}$$

where each matrix element is the absolute difference in phase lag between segment i and segment j. Thus there is no lag w when i = j (lag = 0w), there is one lag between segments when the indices are separated by 1 (lag = 1w), two lags when the indices are separated by 2 (lag = 2w), and three lags when the indices are separated by 3 (lag = 3w). Hence, the contribution toward averaging the autocorrelation function in Eq. 4 is a nonunity weighting of lags, i.e., 0w: 4, 1w: 6, 2w: 4, and 3w: 2. Eq. 4 makes use of only signal segments at lags contained within length N of the signal. By comparison, the autocorrelation function r is computed as the inner product of signal components separated by the same phase lag ϕ:

(6a) $$r\left(w,k\right)=\;\frac{1}{N}\;{\underline{x}}_{N}^{T}\;\cdot \;{\underline{x}}_{N,\;\varphi \;=\;k\cdot w}\;$$

(6b) $$\;=\frac{1}{n\;w}\;\sum_{i}\;{\underline{x}}_{w,i}^{T}\;\cdot \;{\underline{x}}_{w,i+k}\;i=1\;\mathrm{to}\;n$$

where ϕ = kw. In Eq. 6b, the autocorrelation function is described as a summation of inner products of signal segment pairs having length w, with segment ${\underline{x}}_{w,i+k}$ being shifted by k segment lengths (kw sample points) from segment ${\underline{x}}_{w,i}^{T}$. The lags matrix of Eq. 5 shows that the NSE estimator is an unequally weighted average of the autocorrelation function. To include all lags kw, k = 1 to n, so that there is equal weighting when forming an average autocorrelation function, Eq. 6a can be modified as:

(7a) $$\mathrm{rav}\left(w\right)=\frac{1}{\mathrm{nN}}\;\sum_{k}\;{\underline{x}}_{N}^{T}\;\cdot \;{\underline{x}}_{N,\varphi \;=\;k\cdot w}\;k=1\;\mathrm{to}\;n$$

(7b) $$\;=\frac{1}{n^{2}w}\;\sum_{k}\sum_{i}\;{\underline{x}}_{w,i}^{T}\;\cdot \;{\underline{x}}_{w,i+k}\;i=1\;\mathrm{to}\;n,\;k=1\;\mathrm{to}\;n$$

where rav(w) is the autocorrelation function average for all lags ϕ = kw, k = 1 to n, Eq. 7 is computed over an interval **2***N*, and Eq. 7b shows the form when segments of length w are summed. Figure 2 illustrates the process of using this average autocorrelation function as a spectral estimator of CFAE. Firstly, shown in Figure 2A, is the autocorrelation function ${\underline{x}}_{N}^{T}\cdot \;{\underline{x}}_{N,\varphi \;=\;k\cdot w}$, as calculated using Eq. 6. A plot of *r*(*w*,1) versus frequency:

(8) $$f=\frac{\mathrm{sample}\;\mathrm{rate}}{w}$$

is useful as a rough spectral estimator [22-24]. Figure 2A is such a plot except that the abscissa should be converted to a frequency scale using Eq. 8. The process of using the autocorrelation function average as a spectral estimator is shown in Figure 2B. The traces from left to right in the three panels of Figure 2B are constructed using Eq. 7A, with k = 1, 2, and 3, respectively, in the range w = 1 to 500. The abscissa in the respective panels from left to right in Figure 2B has units of lag kw, for 1w, 2w, and 3w. When the three traces in panel B (lags 1w, 2w, 3w), as well as traces for lags 4w, 5w, …, 16w (not shown), are overlapped, the result is depicted in panel C, with the abscissa being in units of Hz (Eq. 8, sample rate 977 Hz). Note that at some frequencies the traces in panel C are reinforced, whereas at other frequencies they are not. When the traces for all lags are summed, as in Eq. 7, the result is shown in Figure 2D, which is the power spectrum based on equally-weighted averaging of the autocorrelation function. The tallest peak occurs at approximately 6.6 Hz, while the second harmonic is at 13.2 Hz and the second subharmonic is at approximately 3.3 Hz. As k increases in Eq. 7, the added component becomes sharper (left to right in Figure 2B), thus contributing fine detail to the spectrum of Figure 2D. This is because a much longer portion kw of the signal, w = 1 to 500, is used for calculation when k is large, with faster falloff away from correlated lags. Where the deflections of individual traces reinforce in panel C, they become spectral peaks in panel D. Where reinforcement occurs, there is a correlation between the signal at most or all lags kw.

To implement Eq. 7 in computer software, the following line of software code can be used:

(9) $$\mathrm{rav}\left(w\right)=\mathrm{rav}\left(w\right)+\;x\left(i\right)\;\cdot \;x\left(i+\mathrm{kw}\right)\;i=1\;\mathrm{to}\;N,\;k=1\;\mathrm{to}\;n$$

where *x*(*i*) is a discrete point in the signal and *x*(*i*+*kw*) is a point, shifted by *i*+*kw* for lags l*w*, 2*w*, …, *nw*. This spectral estimator is normalized as *rav*(*w*)/ *nN* and plotted versus frequency as calculated using Eq. 8, and is termed the AFA estimator.

**Figure 3:**
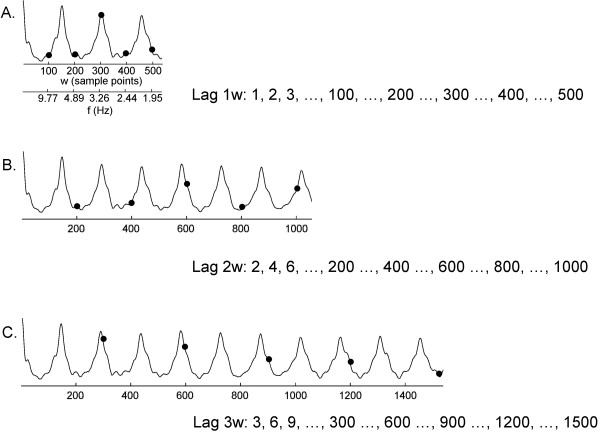
**Construction of lag vectors.** Panels **A-C** show how lag vectors are constructed from the autocorrelation function in the range w = 1 to 500. The solid circles on each graph show the values for w = 100, 200, 300, 400, and 500.

The method for construction of lag vectors which are used to form the AFA estimate is shown in more detail in Figure 3. Lag 1w is depicted in panel A and is the same as Figure 2B, left-hand panel. For reference, the frequency scale for a sampling rate of 977 Hz, as well as the segment length w, is shown on the abscissa. Letting lag 1w range from 1 to 500, corresponding to a frequency of 977 – 1.95 Hz, the lag vector contains 500 points in total. The construction of the lag 2w vector is depicted in panel B. The 500 indices have doubled in value as compared with panel A, and now range from 2 to 1000. Examples of corresponding index numbers for lags 1w and 2w are shown as solid circles in the graphs of panels A and B, respectively. To form the lag 2w vector, the 500 points with lag 2w are extracted from the trace in Figure 3B and plotted, with the result being the middle graph in Figure 2B. This lag vector contributes a sharper set of features to the overall spectral estimate of Figure 2D. Similarly to construct the lag 3w vector, the 500 points with lag 3w are extracted from the trace in Figure 3C and plotted, with the result being the right-hand graph in Figure 2B. This lag vector provides an even sharper contribution to the spectral estimate of Figure 2D. As can be observed in Figure 2A, the autocorrelation function tends to diminish in amplitude with greater lag ϕ, due to small prediction errors. Other investigators have used a damped sinusoidal model for characterization of the autocorrelation function [25], but this is not needed to construct the AFA spectral estimate, as damping and other changes are already built into the lag vectors.

### Introduction of antisymmetry

Although the NSE power spectrum has previously been shown useful for analysis of atrial fractionation, subharmonics and cross-terms are present. To reduce these artifacts, the basis can be made antisymmetric [21]. Consider an ensemble average vector e_w_ with w even, composed of two segments a_w/2_ and b_w/2_, and let all vectors be row vectors:

(10) $${\underline{e}}_{w}\;=\;\left[{\underline{a}}_{w/2}\;{\underline{b}}_{w/2}\right]$$

To impart antisymmetry to the second harmonic, the segments are averaged and the result subtracted from each segment:

(11) $$\begin{array}{l} {\underline{e}}_{w}^{''}\;=\;\left[{\underline{a}}_{w/2}\;{\underline{b}}_{w/2}\right]\;-\;\frac{1}{2}\left[{\underline{a}}_{w/2}\;+\;{\underline{b}}_{w/2}\;{\underline{a}}_{w/2}\;+\;{\underline{b}}_{w/2}\right]\\ \;=\;\frac{1}{2}\;\left[{\underline{a}}_{w/2}\;-\;{\underline{b}}_{w/2}\;\;{\underline{b}}_{w/2}\;-\;{\underline{a}}_{w/2}\right] \end{array}$$

where the double prime symbol ('') indicates that the ensemble vector is now antisymmetric for the second harmonic. To show this, when the two components in Eq. 11 are averaged, they form the 0 vector:

(12) $$\underline{0}=\;\frac{1}{2}\cdot \frac{1}{2}\left[\left({\underline{a}}_{w/2}\;-\;{\underline{b}}_{w/2}\right)\;+\;\left({\underline{b}}_{w/2}\;-\;{\underline{a}}_{w/2}\right)\right]$$

If w is odd, then w/2 will not be an integer and will be subject to rounding error, which will introduce phase noise. To eliminate this drawback would require interpolation between data points so that real numbers are used, which for simplicity is not done in this study.

Impartation of antisymmetry to higher harmonics is commutative and therefore is not order-dependent. This can be shown by numerical example for harmonics 2 and 3. The vectors shown below are row vectors. Consider a signal x:

(13) $$\underline{x}=\left[1\;2\;3\;4\;5\;6\right]$$

The average of the second harmonic is:

(14) $$\langle {\underline{h}}_{2}\rangle =\frac{1}{2}\;\left[1\;2\;3\right]\;+\;\frac{1}{2}\;\left[4\;\;5\;6\right]=\left[\frac{5}{2}\;\;\frac{7}{2}\;\frac{9}{2}\right]$$

Thus:

(15) $${\underline{x}}^{''}=\;\underline{x}\;\;-\;\left[\langle {\underline{h}}_{2}\rangle \;\langle {\underline{h}}_{2}\rangle \right]=\left[-\frac{3}{2}\;-\frac{3}{2}\;-\frac{3}{2}\;\frac{3}{2}\;\frac{3}{2}\;\frac{3}{2}\right]$$

Since ${\underline{x}}^{''}$ is now antisymmetric:

(16) $$\underline{0}=\left[-\frac{3}{2}\;-\frac{3}{2}\;-\frac{3}{2}\right]\;+\;\left[\;\frac{3}{2}\;\frac{3}{2}\;\frac{3}{2}\right]$$

The average of the third harmonic of ${\underline{x}}^{''}$ is:

(17) $$\langle {\underline{h}}_{3}\rangle =\frac{1}{3}\;\left[-\frac{3}{2}\;-\frac{3}{2}\right]\;+\;\frac{1}{3}\;\left[-\frac{3}{2}\;\frac{3}{2}\right]\;+\frac{1}{3}\;\left[\frac{3}{2}\;\frac{3}{2}\right]=\left[-\frac{1}{2}\;\;\frac{1}{2}\right]$$

Thus

(18) $${\underline{x}}^{'\;','\;'\;'}\;={\underline{x}}^{'\;'}-\left[\langle {\underline{h}}_{3}\rangle \;\langle {\underline{h}}_{3}\rangle \;\;\langle {\underline{h}}_{3}\rangle \right]=\left[-1\;-2\;-1\;1\;2\;1\right]$$

Both the second and third harmonics of the new vector are now antisymmetric:

(19) $$\begin{array}{l} \underline{0}\;=\;\left[-1\;-2\;-1\right]\;+\;\left[1\;2\;1\right]\\ \underline{0}\;=\;\left[-1\;-2\right]\;+\;\left[-1\;1\right]\;+\;\left[2\;1\right] \end{array}$$

If the third harmonic of x is first made antisymmetric:

(20) $$\langle {\underline{h}}_{3}\rangle =\frac{1}{3}\;\left[1\;2\right]\;+\;\frac{1}{3}\;\left[3\;\;4\right]\;\;+\;\frac{1}{3}\;\left[5\;\;6\right]=\left[3\;4\right]$$

Then:

(21) $$\begin{array}{l} {\underline{x}}^{'\;'\;'}=\underline{x}\;-\;\left[\langle {\underline{h}}_{3}\rangle \;\langle {\underline{h}}_{3}\rangle \;\langle {\underline{h}}_{3}\rangle \right]\\ =\left[1\;2\;3\;4\;5\;6\right]\;-\;\left[3\;4\;3\;4\;3\;4\right]\\ =\left[-2\;-2\;0\;0\;2\;2\right] \end{array}$$

If the second harmonic of ${\underline{x}}^{'\;'\;'}$ is then made antisymmetric:

(22) $$\langle {\underline{h}}_{2}\rangle \;=\;\frac{1}{2}\;\left[-2\;-2\;0\right]\;+\;\frac{1}{2}\;\left[0\;\;2\;\;2\right]=\left[-1\;0\;1\right]$$

Then:

(23) $${\underline{x}}^{'\;'\;',\;'\;'}={\underline{x}}^{'\;'\;'}-\;\left[\langle {\underline{h}}_{2}\rangle \;\langle {\underline{h}}_{2}\rangle \right]=\left[-1\;-2\;-1\;1\;2\;1\right]$$

where the right hand sides of Eq.’s 18 and 23 are identical. Therefore, the order of harmonic removal is inconsequential, and antisymmetry is maintained for each harmonic that is removed. If the basis contains little or no power in the second and third harmonics, then $\langle {\underline{h}}_{2}\rangle$, $\langle {\underline{h}}_{3}\rangle$ … will trend toward zero vectors, and there will be no significant change in the shape of the original ensemble vector nor change in its power by imparting antisymmetry.

**Figure 4:**
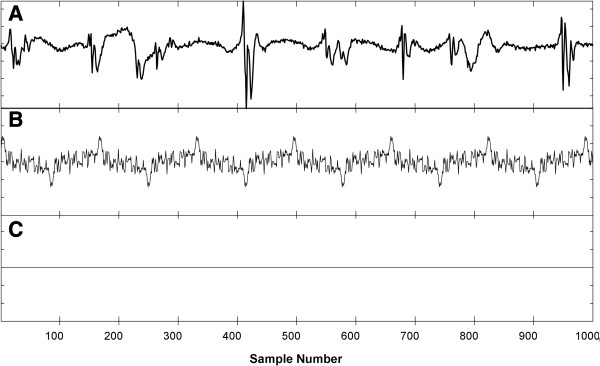
**Example of removal of harmonics from the ensemble average of the DF, which is done for the NSH estimator implementation.** Panel **A**: Original signal. Panel **B**: The ensemble average of the dominant frequency with antisymmetry imparted to the second harmonic. Panel **C**: Addition of the antisymmetric components of the ensemble average in panel B results in the zero vector.

In Figure 4 is shown an example of harmonic removal. A recording from the left superior pulmonary vein, persistent AF patient is shown in panel A for 1000 sample points. For this signal the DF is 5.96 Hz (w = 164). The ensemble average at the DF after antisymmetry is imparted to the second harmonic is shown repeated in panel B. Addition of the two antisymmetric components, each of length 164 / 2 = 82 points, forms the zero vector, shown in panel C. When antisymmetry is imparted to the ensemble averages prior to power spectral calculation, the resulting spectral estimator is termed the novel spectral estimator with harmonics removed (NSH).

### Implementation of estimators and spectral measurements

The aforementioned spectral estimators were compared to the Fourier power spectrum, which was computed using a radix-2 implementation of the DFT [26]. For ease of comparison, rectangular windowing was used for all estimators, so that frequency resolution was not diminished [20]. Furthermore, for better comparison, the preprocessing step of bandpass filtering, signal rectification, and low pass filtering [12,13] was not implemented. For calculation of spectral parameters, for simplicity the NSH estimator was implemented with antisymmetry imparted to the second harmonic only, where most of the harmonic power resides.

The following parameters were used for comparison of the spectral estimators in the physiologic range of 3-12 Hz. The DF was determined by automatically selecting the tallest spectral peak in the range 3.5-8.5 Hz [21] with manual correction when necessary if the fundamental frequency was found to reside between 3–3.5 Hz or between 8.5-12 Hz (in practice it is almost always found between 3.5-8.5 Hz). This practical implementation solves the problem of inadvertently selecting a subharmonic as the DF during the automatic detection process, as was done in a previous study [27]. The dominant amplitude (DA) was also measured, defined as the amplitude of the DF peak [21]. A larger value of DA indicates that more signal power resides in the dominant periodic component, as can occur when a stable local electrical activation pattern is the source. The magnitude of the power spectrum from 3-12 Hz was then normalized to a range of 0–1 units. The mean of this normalized spectral profile (MP), which is indicative of the spectral baseline level and therefore the degree of global electrical activation pattern stability [21], was also calculated.

### Comparison of fast activation rate versus highly fractionated CFAE

As a further check of the methods, the CFAE were then subdivided according to morphologic differences. All CFAE were first scaled to a peak-to-peak value of 1.0 millivolts. Fast activation rate CFAE were defined as those scaled electrograms with a standard deviation < 0.074. Highly fractionated CFAE were defined as those scaled electrograms with a standard deviation ≥ 0.074. The threshold was taken so as to subdivide the number of CFAE in each subgroup evenly (102 electrograms in each group). This quantitative definition corresponded to the visual appearance of these electrograms. For each of the two subgroups, the same spectral parameters were compared for paroxysmal versus persistent CFAE using all four estimators.

### Statistical calculations

The DFT, NSE, NSH, and AFA estimators were compared statistically based on the three measured spectral parameters. The Mann–Whitney Rank Sum Test, a nonparametric procedure which does not require assumption of normality or equal variance, was used to determine significant differences in the means in the spectral parameters, and the F-test was used to determine significant differences in the standard deviations (SigmaPlot 2004 for Windows Ver. 9.01, Systat Software, Chicago, MedCalc ver. 9.5, 2008, MedCalc Software bvba, Mariakerke, Belgium). Differences were considered significant at a level p < 0.05.

## Results

**Figure 5:**
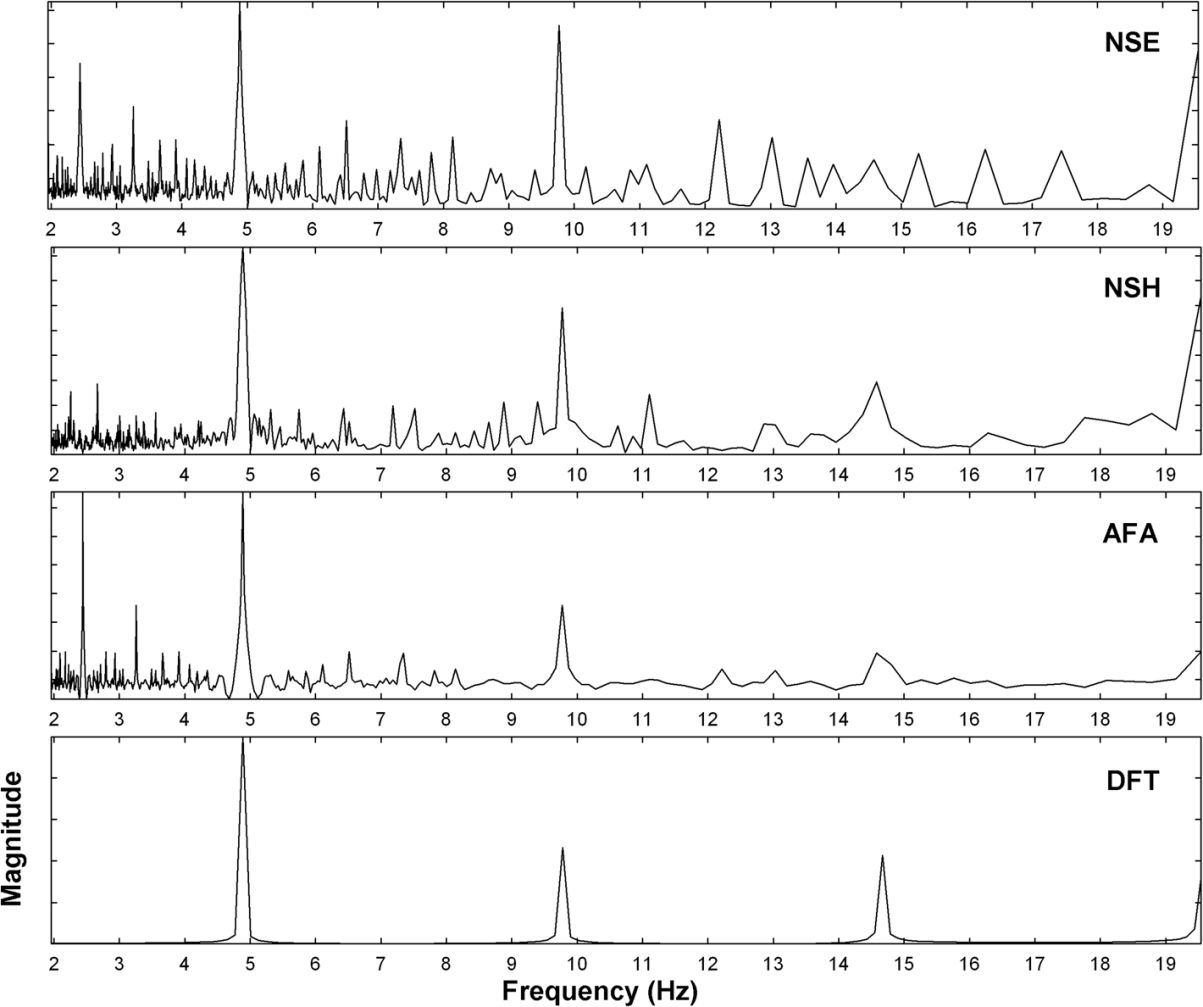
**Examples of power spectra for the four estimators.** The spectra are shown in the range 2 – 20 Hz for clarity.

An example of the power spectra for each of the estimators using a repetitive electrogram pattern having a frequency of 4.89 Hz is shown in Figure 5. This example was used to show that each estimator could detect the DF. From panels A to D are spectra from NSE, NSH, AFA, and DFT estimators. The frequency range is 2 – 20 Hz to show presence of sub- and superharmonics. For all spectra, the tallest peak in the electrophysiologic range of 3 – 12 Hz, the DF, is at 4.89 Hz, the frequency of the repetitive pattern. The 2.45 Hz subharmonic is evident in NSE and AFA spectra. Cross-terms are also present in the NSE spectrum, for example at 3.26 Hz (2/3 integer relationship with the DF) and 12.23 Hz (5/2 integer relationship with the DF). Subharmonics and cross-terms are mostly absent from the NSH spectrum, which for illustrative purposes was constructed using antisymmetric bases for the 2nd, 3rd, 5th, and 7th harmonics.

### Summary statistics

Measurements for all data are shown in Tables 1, 2 and 3. The DA spectral parameter results are summarized in Table 1. For all measurement methods, mean DA was greater in persistent as compared with paroxysmal AF. This was highly significant for the NSE and NSH estimators (p < 0.001). The standard deviation in the DA spectral parameter was greater in persistent AF as compared with paroxysmal AF for all methods. This was highly significant (p < 0.001), except for the DFT estimator which was not significant. For all measurement methods, the mean DF was also greater in persistent AF as compared with paroxysmal AF (Table 2). This was highly significant for all methods (p < 0.001). The standard deviation in DF was larger in paroxysmals for all methods, which achieved moderate significance only for NSE and NSH (p < 0.05). Measurements for the MP spectral parameter are shown in Table 3. For all methods, mean MP was greater in paroxysmal as compared with persistent AF patients. The differences in mean MP were highly significant for all four methods (p < 0.001). The differences in the standard deviation of MP were highly significant for the NSE and NSH estimators (p ≤ 0.001).

**Table 1:** Dominant amplitude

**Method**	**MN - Par**	**SD - Par**	**MN - Per**	**SD – Per**	**MN Signif**	**SD Signif**
NSE	1.472	0.231	1.839	0.604	p < 0.001	p < 0.001
NSH	1.192	0.225	1.521	0.483	p < 0.001	p < 0.001
AFA	0.141	0.172	1.173	4.637	p = 0.140	p < 0.001
DFT	0.688	0.297	0.849	0.375	p = 0.004	p = 0.064

*Par* paroxysmal AF, *Per* persistent AF, *MN* mean, *SD* standard deviation, *Signif* significance. For DA and DF, only pulmonary vein ostia recordings were used and N = 60 and 76, respectively, for paroxysmal versus persistent CFAE. For MP, all sites were used and N = 90 and 114, respectively, for paroxysmal versus persistent CFAE.

**Table 2:** Dominant frequency

**Method**	**MN - Par**	**SD - Par**	**MN - Per**	**SD - Per**	**MN Signif**	**SD Signif**
NSE	5.741	1.235	6.196	0.969	p < 0.001	p = 0.047
NSH	5.732	1.274	6.420	0.977	p < 0.001	p = 0.030
AFA	5.317	1.182	5.962	1.176	p < 0.001	p = 0.959
DFT	5.623	1.126	6.253	0.919	p < 0.001	p = 0.096

*Par* paroxysmal AF, *Per* persistent AF, *MN* mean, *SD* standard deviation, *Signif* significance. For DA and DF, only pulmonary vein ostia recordings were used and N = 60 and 76, respectively, for paroxysmal versus persistent CFAE. For MP, all sites were used and N = 90 and 114, respectively, for paroxysmal versus persistent CFAE.

**Table 3:** Mean spectral profile

**Method**	**MN - Par**	**SD - Par**	**MN – Per**	**SD - Per**	**MN Signif**	**SD Signif**
NSE	0.401	0.062	0.337	0.104	p < 0.001	p < 0.001
NSH	0.375	0.069	0.308	0.096	p < 0.001	p = 0.001
AFA	0.358	0.088	0.304	0.099	p < 0.001	p = 0.247
DFT	0.305	0.068	0.258	0.071	p < 0.001	p = 0.674

*Par* paroxysmal AF, *Per* persistent AF, *MN* mean, *SD* standard deviation, *Signif* significance. For DA and DF, only pulmonary vein ostia recordings were used and N = 60 and 76, respectively, for paroxysmal versus persistent CFAE. For MP, all sites were used and N = 90 and 114, respectively, for paroxysmal versus persistent CFAE.

Overall, the NSE and NSH methods had highly significant differences in the means for DA, DF, and MP (p<0.001). The AFA and DFT methods had highly significant differences in the means for DA and MP only (p<0.001). Overall, NSE and NSH had highly significant differences in the standard deviations for DA and MP (p<0.001) and moderately significant difference for DF (p<0.05)s. AFA had a highly significant difference in standard deviation for the DA measurement. The DFT had no significant differences in standard deviation for any of these measurements. Thus based on significant differences in spectral estimation measurements between paroxysmal and persistent AF, the NSE and NSF methods had greatest efficacy, followed by AFA and DFT.

Tables 4, 5 and 6 show the results for fast activation rate CFAE only. Similar to Tables 1, 2 and 3, there are higher values for DA and DF in persistent AF for all estimators, and higher values of MP in paroxysmal AF for all estimators. The NSE and NSH estimators exhibit significant differences in paroxysmals versus persistents for all three spectral parameters. The AFA and DFT estimators exhibit significant differences in paroxysmals versus persistents for only the DF spectral parameter. Tables 7, 8 and 9 show the results for highly fractionated CFAE only. Similar to Tables 1, 2 and 3 and Tables 4, 5 and 6, there are higher values for DA and DF in persistent AF for all estimators, and higher values of MP in paroxysmal AF for all estimators. The NSE, NSH, and DFT estimators exhibit significant differences in paroxysmals versus persistents for two of three spectral parameters. The AFA estimator exhibits significant differences in paroxysmals versus persistents for one of three spectral parameters – the MP. For both subgroups (Tables 4, 5 and 6 and Tables 7, 8 and 9), overall there were significant differences in standard deviations at the p<0.01 level in 5/6 comparisons for the NSE and NSH estimators, in 2/6 comparisons for the AFA estimator, and in 1/6 comparisons for the DFT estimator.

**Table 4:** Fast activation rate - dominant amplitude

**Method**	**MN - Par**	**SD – Par**	**MN - Per**	**SD - Per**	**MN Signif**	**SD Signif**
NSE	1.453	0.210	1.679	0.442	P = 0.008	P < 0.001
NSH	1.175	0.208	1.411	0.415	P = 0.003	P < 0.001
AFA	0.012	0.016	0.106	0.563	P = 0.412	P < 0.001
DFT	0.688	0.299	0.741	0.368	P = 0.600	P = 0.099

*Par* paroxysmal AF, *Per* persistent AF, *MN* mean, *SD* standard deviation, *Signif* significance.

**Table 5:** Fast activation rate - dominant frequency

**Method**	**MN - Par**	**SD - Par**	**MN - Per**	**SD - Per**	**MN Signif**	**SD Signif**
NSE	5.689	1.225	6.352	1.031	P = 0.003	P = 0.158
NSH	5.545	1.337	6.665	0.949	p < 0.001	P = 0.005
AFA	5.028	1.025	6.004	1.229	p < 0.001	P = 0.148
DFT	5.444	1.068	6.454	0.982	p < 0.001	P = 0.489

*Par* paroxysmal AF, *Per* persistent AF, *MN* mean, *SD* standard deviation, *Signif* significance.

**Table 6:** Fast activation rate - mean spectral profile

**Method**	**MN – Par**	**SD – Par**	**MN - Per**	**SD - Per**	**MN Signif**	**SD Signif**
NSE	0.400	0.057	0.352	0.091	P = 0.004	P < 0.001
NSH	0.382	0.062	0.319	0.089	p < 0.001	P < 0.001
AFA	0.369	0.087	0.340	0.086	P = 0.094	P = 0.902
DFT	0.307	0.076	0.277	0.066	P = 0.054	P = 0.156

*Par* paroxysmal AF, *Per* persistent AF, *MN* mean, *SD* standard deviation, *Signif* significance.

**Table 7:** Highly fractionated - dominant amplitude

**Method**	**MN - Par**	**SD – Par**	**MN - Per**	**SD - Per**	**MN Signif**	**SD Signif**
NSE	1.509	0.270	2.037	0.716	p < 0.001	P < 0.001
NSH	1.226	0.259	1.658	0.530	p < 0.001	P < 0.001
AFA	0.027	0.025	0.185	0.555	P = 0.084	P < 0.001
DFT	0.688	0.301	0.984	0.343	p < 0.001	P = 0.298

*Par* paroxysmal AF, *Per* persistent AF, *MN* mean, *SD* standard deviation, *Signif* significance.

**Table 8:** Highly fractionated - dominant frequency

**Method**	**MN - Par**	**SD – Par**	**MN - Per**	**SD - Per**	**MN Signif**	**SD Signif**
NSE	5.845	1.281	6.003	0.863	P = 0.591	P = 0.001
NSH	6.106	1.073	6.118	0.939	P = 0.936	P = 0.274
AFA	5.896	1.285	5.910	1.122	P = 0.781	P = 0.266
DFT	5.981	1.181	6.005	0.778	P = 0.914	P = 0.001

*Par* paroxysmal AF, *Per* persistent AF, *MN* mean, *SD* standard deviation, *Signif* significance.

**Table 9:** Highly fractionated - mean spectral profile

**Method**	**MN - Par**	**SD – Par**	**MN - Per**	**SD - Per**	**MN Signif**	**SD Signif**
NSE	0.401	0.068	0.323	0.113	p < 0.001	P < 0.001
NSH	0.368	0.076	0.297	0.101	p < 0.001	P = 0.006
AFA	0.345	0.088	0.272	0.099	p < 0.001	P = 0.247
DFT	0.302	0.057	0.240	0.071	p < 0.001	P = 0.031

*Par* paroxysmal AF, *Per* persistent AF, *MN* mean, *SD* standard deviation, *Signif* significance.

## Discussion

### Summary

In this study, novel methods of spectral estimation were compared to traditional Fourier analysis for characterization of complex fractionated electrograms recorded during atrial fibrillation. The foundation for these novel techniques is a recently described transform in which the power spectrum is computed by averaging the autocorrelation function at lags [19]. Since the DFT estimator models the autocorrelation function using a sinusoidal approximation (Wiener–Khinchin theorem), all four spectral estimators are derived from the autocorrelation function. Three spectral parameters were used to test the efficacy of each spectral estimator. Along with the DF which is commonly used, two additional spectral measurements, the DA and MP, were also calculated. Efficacy was determined from the significance of the difference in the means and standard deviations of the measurements when comparing paroxysmal versus persistent AF data. Two of the methods, NSE and NSH, were significantly improved with respect to the DFT for discerning CFAE spectral parameters in paroxysmal versus persistent AF patients. Although the AFA method, calculated over a signal length 2N, provided only marginal improvement as an estimator as compared with the DFT, it is simple to implement, requiring only one line of software code to describe the calculation (Eq. 9).

### Clinical correlates

Although the DF is often used for frequency analysis of CFAE, our study suggests that other spectral parameters may be more useful for fractionated atrial electrogram analysis. The DA parameter is related to DF power, and is therefore somewhat similar to the regularity index sometimes used in previous studies [14,28]. The regularity index is defined as the power of the dominant peak divided by the overall spectral power. However the regularity index requires the width of the dominant peak to be guesstimated. Furthermore, the regularity index is normalized over an arbitrary power spectral range. No such guesstimates and arbitrary impositions are used for DA calculation, so that it is potentially a more robust measure for clinical application. The MP parameter was measured over the accepted electrophysiologic range of 3-12 Hz [21]. It is therefore a more global spectral measure as compared with the DA and DF parameters, and as such is more similar to the organizational index described in previous studies [14,28]. The organizational index is defined as the spectral power of the DF and its harmonics divided by the overall spectral power. The organizational index however, involves guestimation of the width of the dominant peak, as well as the width of harmonics whose power should be summed, and the number of harmonics to be summed. In contrast, the MP spectral parameter does not use guesstimates, so that it is likely to be a more robust measure for clinical application.

The NSE transform for spectral estimation and signal reconstruction has also been applied to other applications including ventricular tachyarrhythmias [29] and to videocapsule imagery data in celiac disease patients [30]. The method is therefore likely applicable to a variety of data types. The spectral parameters described in this study have also been used for QRST cancellation [31] and the NSE estimator has been incorporated into a study for heart sounds quantification [32]. Heart sounds patterns have also been detected by averaging segments of the acoustic signal at different lengths w, similar to the NSE implementation [33].

### Limitations

The General Electric CardioLab acquisition settings are hardwired for signal digitization at a rate of 977 Hz after antialiasing with a single pole filter having a 500 Hz corner frequency. These settings cause any high frequency components from 489 – 500 Hz to pass through the filter without sufficiently fast digitization for accurate representation, a limitation of the system. The study was done using retrospective data and with a relatively small number of patient recordings. The results should be confirmed in a larger, prospective study.

## Conclusions

Measurements made from all four spectral estimators used in the study were in agreement as to whether the means and standard deviations in three spectral parameters were greater in paroxysmal or in persistent AF. Since the measurements were consistent, implementation of several of these estimators for power spectral analysis should be useful for verification of the findings. Since the most significant differences overall were achieved using the NSE and NSH estimators, parameters measured from their spectra will likely be the most helpful for detecting and discerning electrophysiologic differences in the AF substrate. For all methods, to varying degrees of significance, the DP parameter was greater and the DF higher in persistent AF, while the MP was larger in paroxysmal AF, likely reflecting a greater uniformity in remodeling and greater stability of the activation pattern in the arrhythmogenic substrate of persistent AF patients.

## Abbreviations

CFAE: Complex fractionated atrial electrograms; DFT: Discrete Fourier transform; NSE: Novel spectral estimator; NSH: NSE with harmonic removal; AFA: Autocorrelation function average at lags; DF: Dominant frequency; DA: Dominant amplitude; MP: Mean spectral profile.

## Competing interests

The authors declare that they have no competing interests.

## Authors’ contributions

EJC did the quantitative work and wrote the manuscript. AB and HG acquired the clinical data, selected CFAE for analysis, and reviewed the manuscript. All authors read and approved the final manuscript.
